# Pathologic complete response in patients with esophageal cancer receiving neoadjuvant chemotherapy or chemoradiation: A systematic review and meta‐analysis

**DOI:** 10.1002/cam4.7076

**Published:** 2024-03-08

**Authors:** Charles E. Gaber, Jyotirmoy Sarker, Abdullah I. Abdelaziz, Ebere Okpara, Todd A. Lee, Samuel J. Klempner, Ryan D. Nipp

**Affiliations:** ^1^ Department of Pharmacy Systems, Outcomes and Policy, College of Pharmacy University of Illinois Chicago Chicago Illinois USA; ^2^ Center for Pharmacoepidemiology and Pharmacoeconomic Research, College of Pharmacy University of Illinois Chicago Chicago Illinois USA; ^3^ Massachusetts General Hospital Cancer Center Boston Massachusetts USA; ^4^ OU Health Stephenson Cancer Center Oklahoma University Oklahoma City Oklahoma USA

**Keywords:** chemotherapy, esophageal cancer, meta‐analysis, neoadjuvant chemoradiation, pathologic complete response

## Abstract

**Background:**

Neoadjuvant chemoradiation and chemotherapy are recommended for the treatment of nonmetastatic esophageal cancer. The benefit of neoadjuvant treatment is mostly limited to patients who exhibit pathologic complete response (pCR). Existing estimates of pCR rates among patients receiving neoadjuvant therapy have not been synthesized and lack precision.

**Methods:**

We conducted an independently funded systematic review and meta‐analysis (PROSPERO CRD42023397402) of pCR rates among patients diagnosed with esophageal cancer treated with neoadjuvant chemo(radiation). Studies were identified from Medline, EMBASE, and CENTRAL database searches. Eligible studies included trials published from 1992 to 2022 that focused on nonmetastatic esophageal cancer, including the gastroesophageal junction. Histology‐specific pooled pCR prevalence was determined using the Freeman–Tukey transformation and a random effects model.

**Results:**

After eligibility assessment, 84 studies with 6451 patients were included. The pooled prevalence of pCR after neoadjuvant chemotherapy in squamous cell carcinomas was 9% (95% CI: 6%–14%), ranging from 0% to 32%. The pooled prevalence of pCR after neoadjuvant chemoradiation in squamous cell carcinomas was 32% (95% CI: 26%–39%), ranging from 8% to 66%. For adenocarcinoma, the pooled prevalence of pCR was 6% (95% CI: 1%–12%) after neoadjuvant chemotherapy, and 22% (18%–26%) after neoadjuvant chemoradiation.

**Conclusions:**

Under one‐third of patients with esophageal cancer who receive neoadjuvant chemo(radiation) experience pCR. Patients diagnosed with squamous cell carcinomas had higher rates of pCR than those with adenocarcinomas. As pCR represents an increasingly utilized endpoint in neoadjuvant trials, these estimates of pooled pCR rates may serve as an important benchmark for future trial design.

## Graphical abstract

This systematic review and meta‐analysis revealed that less than one‐third of nonmetastatic esophageal cancer patients achieve pathologic complete response (pCR) after neoadjuvant chemoradiation or chemotherapy, with squamous cell carcinomas exhibiting higher pCR rates than adenocarcinomas. These findings derived from a robust pool of 84 studies could offer a critical benchmark for future trial design.

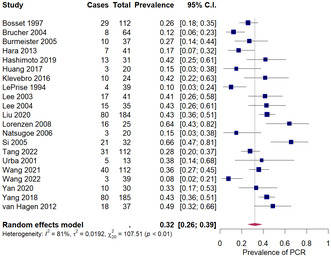

## 1 INTRODUCTION

Esophageal cancer is a leading cause of global cancer‐specific mortality, with less than 20% of individuals surviving 5 years of past diagnosis.^1^ Esophageal cancer accounts for over 16,000 deaths in the United States annually, with the burden of disease expected to increase over time as the population ages.^2^, ^3^ At the time of diagnosis, a plurality of individuals present with advanced disease.^4^ Landmark clinical trials over the past several decades have demonstrated that neoadjuvant therapy prior to esophagectomy confers a significant survival advantage over surgery alone.^5^, ^6^, ^7^ Consequently, current treatment guidelines reflect these findings, recommending several neoadjuvant options for treating locally advanced tumors: neoadjuvant chemotherapy and/or chemoradiation followed by esophagectomy and perioperative chemotherapy.^8^, ^9^, ^10^

Improved survival and quality of life are key considerations when selecting treatment for esophageal cancer.^11^ To nominate promising neoadjuvant therapeutic strategies, pathologic response in the primary tumor is a relatively rapid readout proposed as a surrogate for antitumor activity. Research has demonstrated that pathologic complete response (pCR) following neoadjuvant therapy often correlates with improved recurrence‐free and overall survival.^12^, ^13^, ^14^, ^15^, ^16^, ^17^, ^18^, ^19^, ^20^ However, most patients do not experience pCR following neoadjuvant therapy, and individuals receiving trimodality therapy without response to chemoradiation have been shown to have survivals approximating surgery alone.^21^, ^22^ Currently, no studies provide pooled, durable estimates of the expected pCR rate for patients with esophageal cancer receiving neoadjuvant therapy. While multiple trials have reported on this outcome, individually they are small studies with limited precision and do not examine how patient factors may predict likelihood of experiencing pCR.

In this study, we performed a systematic review and meta‐analysis to summarize the existing literature on pCR amongst individuals with nonmetastatic esophageal cancer who received either neoadjuvant chemotherapy and/or chemoradiation. We aimed to characterize the associations between certain study‐ and patient‐level factors and the outcome of pCR. Procuring precise estimates of the probability of experiencing pCR after neoadjuvant therapy can help to inform the design of trials to more efficiently prioritize new agents in esophageal cancer.

## 2 MATERIALS AND METHODS

### 2.1 Study identification

We performed a literature review in accordance with the guidance established by the Preferred Reporting Items for Systematic Reviews and Meta‐Analyses (PRISMA) 2020 statement^23^ and the JBI methodology for systematic reviews of prevalence and incidence.^24^ Table S1 contains the PRISMA checklist. We identified relevant studies in the following three electronic databases: (1) Medline; (2) EMBASE; and (3) Cochrane Central Register of Controlled Trials (CENTRAL). Table S2 contains the search strategy used for study identification in each database, implemented on November 30, 2022. We pre‐registered the review protocol on PROSPERO (CRD42023397402) before eligibility assessment and data extraction occurred (but after the search was performed and locked‐in). We utilized the Covidence web‐based platform for systematic review data management.

### 2.2 Eligibility criteria

Prospective clinical trials (randomized, single arm, and nonrandomized) of individuals with incident, nonmetastatic esophageal carcinoma of any histology, including all sites of the esophagus and the gastroesophageal junction (GEJ), that contained at least one trial treatment arm of neoadjuvant therapy were eligible for inclusion. Nonrandomized trials were included because they constitute an interventional study design and the summary measure of our analysis (prevalence of pCR) was not being compared across arms. Neoadjuvant therapies consisted of chemotherapy (including perioperative) and chemoradiation (including induction chemotherapy followed by chemoradiation). We focused on cytotoxic chemotherapies; we did not include HER‐2 targeted therapy. Thus, if neoadjuvant chemo(radiation) strategies were coupled with other neoadjuvant treatment types, such as immunotherapy, they were excluded because we wanted to isolate the effect of only guideline‐recommended treatment strategies on pCR. However, studies could have nonchemotherapy adjuvant treatment. A minimum arm size of 20 individuals receiving resection after neoadjuvant therapy for evaluation of pCR was required to ensure inclusion of well‐powered studies. Eligible studies were required to report the prevalence of pCR in patients receiving neoadjuvant therapy. Several methods for evaluating pCR exist, such as the Mandard^25^ and Chirieac^19^ tumor regression grade (TRG) systems. All systems were eligible for inclusion, but if a study did not specify a TRG, we required the trial to have either explicitly labeled their response outcome as pCR or the absence of residual disease upon pathology. Studies published in English between 1992 and 2022 from any geographic location were eligible.

### 2.3 Study selection

After implementing the protocol search strategy in the three databases and de‐duplicating records, the titles and abstracts of all search results were screened for relevance by two independent reviewers (JS and AA). Following screening, the full‐text publications for the screen‐eligible studies were reviewed for further evaluation of eligibility by two of three potential independent reviewers (JS, AA, and EO). Any disagreement about eligibility (screening stage or full‐text stage) was resolved by a third investigator (CG).

### 2.4 Assessment of methodologic quality

As our study focused on pooling a percentage (rather than a contrast measure such as hazard ratio of relative risk), there was not “between‐arms” bias such as confounding to evaluate with standard risk‐of‐bias tools. Thus, we critically appraised the methodologic quality of eligible studies using a study‐specific adaptation of the JBI Critical Appraisal Checklist for Studies Reporting Prevalence Data.^24^ The adapted JBI checklist contained five items for assessment (Table S2). Studies had their data extracted and included in the final analysis regardless of their assessed quality, but quality assessments for each study are included in Table S3.

### 2.5 Data extraction

Study‐level fields were extracted by a single reviewer and consisted of authorship, year of publication, geographic location(s) where the trial was performed, and number of patients stratified by histologic subtype. Within studies, treatment arm‐specific fields extracted included neoadjuvant treatment class (nCT or nCRT), specific chemotherapy agents used, and pCR rate. Number of patients and pCR rate were stratified according to histologic subtype if the trial included both adenocarcinoma and squamous cell carcinoma and reported histology‐specific pCR rates. Data extracted from studies are available as supplemental materials.

### 2.6 Statistical analysis

We calculated the pooled prevalence of pCR, overall and according to tumor histology and type of neoadjuvant therapy. Neoadjuvant chemotherapy and perioperative chemotherapy were analyzed together given that chemotherapy was the only treatment delivered prior to surgery and pathological response assessment. Likewise, neoadjuvant chemoradiation and induction chemotherapy with chemoradiation were analyzed together. We used the Freeman–Tukey double arcsine transformation to calculate the pooled prevalence using a random‐effects model with inverse‐variance weighting calculated via the DerSimonian and Laird method. Results were visually displayed using forest plots. We used univariable meta‐regression models to assess whether the pCR rate was associated with patient and treatment characteristics.

## 3 RESULTS

**FIGURE 1:**
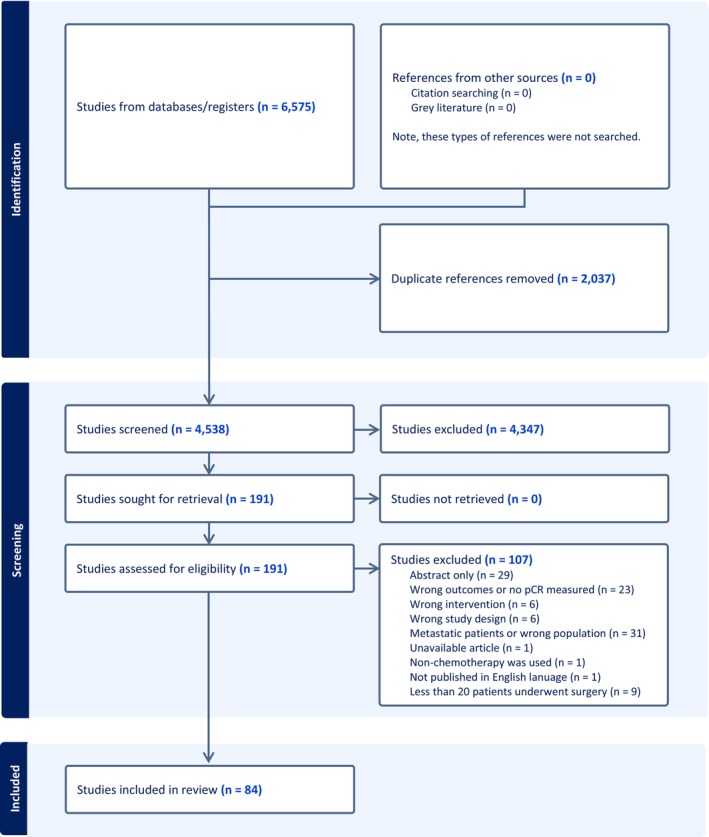
PRISMA 2020 diagram depicting identification, screening, and inclusion of studies.

We retrieved 6575 records from the preliminary database searches (Figure 1). After elimination of 2037 duplicate records, we then screened the titles and abstracts of 4538 records, 4347 of which were excluded due to not meeting eligibility criteria. The full‐text publications for the remaining 191 studies were reviewed. Of these, 84 studies^7^, ^26^, ^27^, ^28^, ^29^, ^30^, ^31^, ^32^, ^33^, ^34^, ^35^, ^36^, ^37^, ^38^, ^39^, ^40^, ^41^, ^42^, ^43^, ^44^, ^45^, ^46^, ^47^, ^48^, ^49^, ^50^, ^51^, ^52^, ^53^, ^54^, ^55^, ^56^, ^57^, ^58^, ^59^, ^60^, ^61^, ^62^, ^63^, ^64^, ^65^, ^66^, ^67^, ^68^, ^69^, ^70^, ^71^, ^72^, ^73^, ^74^, ^75^, ^76^, ^77^, ^78^, ^79^, ^80^, ^81^, ^82^, ^83^, ^84^, ^85^, ^86^, ^87^, ^88^, ^89^, ^90^, ^91^, ^92^, ^93^, ^94^, ^95^, ^96^, ^97^, ^98^, ^99^, ^100^, ^101^, ^102^, ^103^, ^104^, ^105^, ^106^, ^107^, ^108^ met the inclusion and exclusion criteria and were included in this systematic review and meta‐analysis, yielding a total of 6451 patients who received neoadjuvant therapy and had tumor response measured pathologically after surgery. Of the 84 included trials, 13 (15%) were published between 1992 and 2001, 31 (37%) between 2002 and 2011, and 41 (49%) between 2012 and 2022. In terms of study design, 44 (52%) of the included studies were single‐arm trials, 38 (45%) were randomized trials, and three (4%) were nonrandomized trials of multiple treatments. Histologically, 32 (38%) studies were performed amongst study populations with squamous cell carcinoma only, 15 (18%) in adenocarcinoma only, 26 (31%) in squamous cell carcinoma and adenocarcinoma, and 12 (14%) included other histologic subtypes along with squamous cell and adenocarcinoma. Geographically, 32 (38%), 28 (33%), 23 (27%), and 2 (2%) of the included trials were performed in Asia, Europe, North America, and Australia, respectively. Across 102 trial arms that delivered either neoadjuvant chemotherapy or chemoradiation, platinum, and fluorouracil‐based regimens were the most common *(n* = 41, 40%), followed by platinum and taxane‐based regimens (*n* = 28, 27%), regimens that contained platinum‐based agents, fluorouracil, and a taxane (*n* = 16, 16%), and other regimens (*n* = 17, 17%). The full distribution of regimens is presented in Figure S1.

### 3.1 Pathologic complete response

**FIGURE 2:**
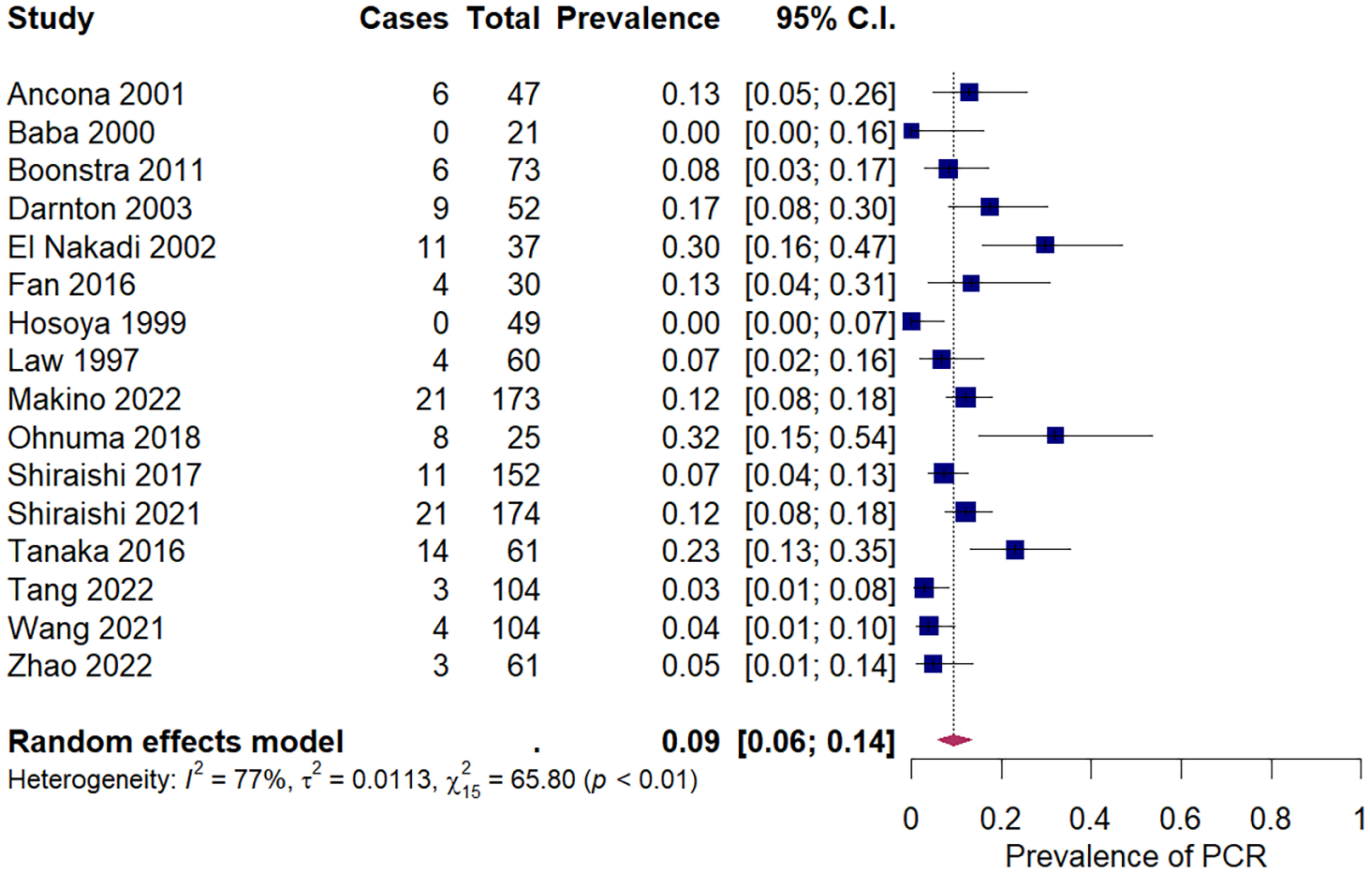
Pathologic complete response amongst squamous cell carcinoma patients receiving neoadjuvant chemotherapy.

**FIGURE 3:**
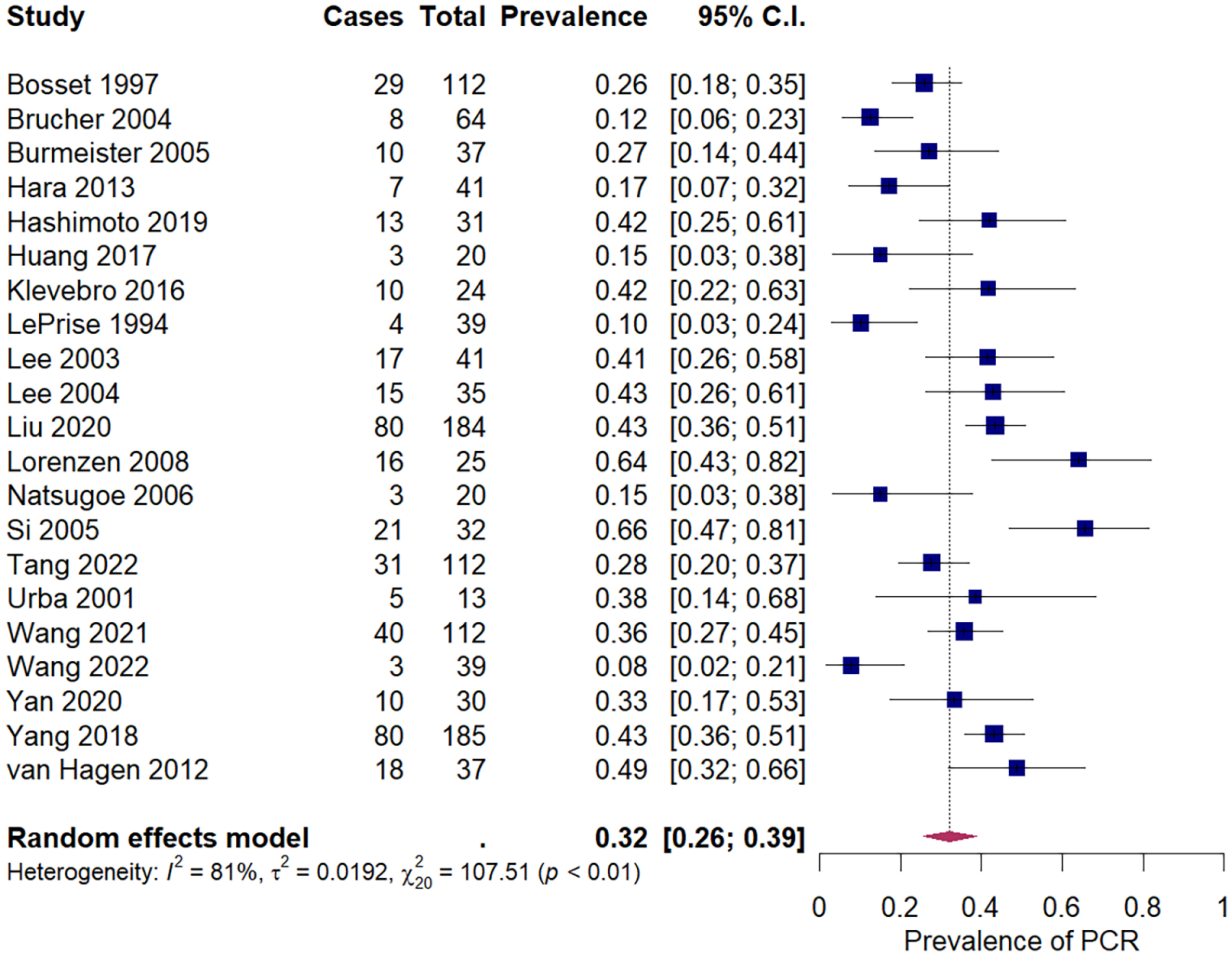
Pathologic complete response amongst squamous cell carcinoma patients receiving neoadjuvant chemoradiation.

**FIGURE 4:**
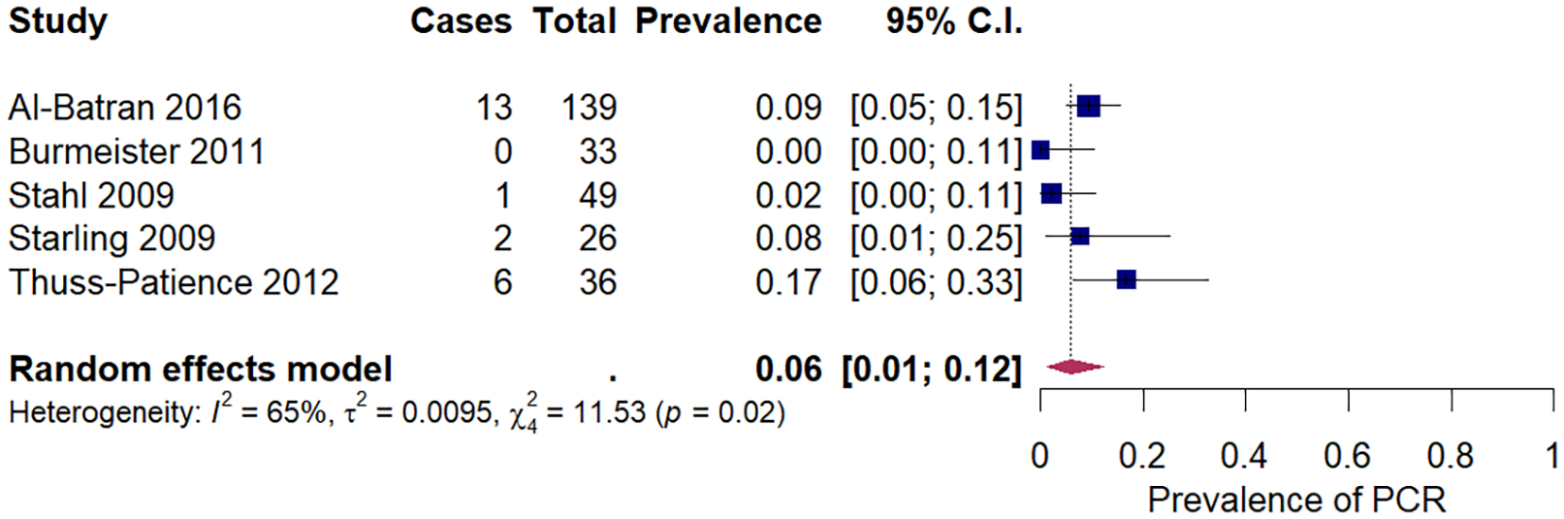
Pathologic complete response amongst adenocarcinoma patients receiving neoadjuvant chemotherapy.

**FIGURE 5:**
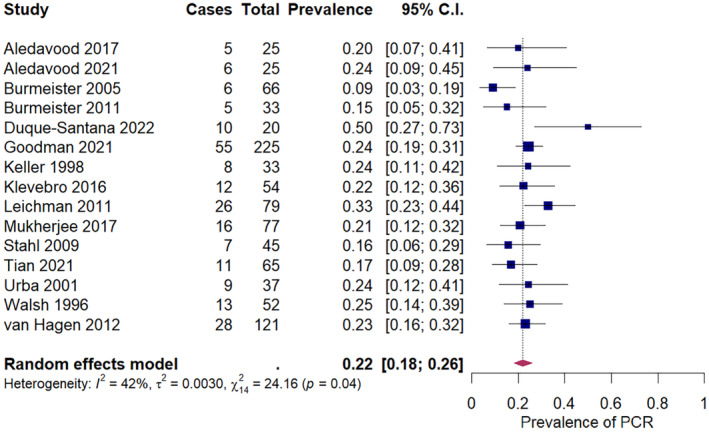
Pathologic complete response amongst adenocarcinoma patients receiving neoadjuvant chemoradiation.

The pooled prevalence of pCR after neoadjuvant chemotherapy in squamous cell carcinoma was 9% (95% CI: 6%–14%) across 16 studies, ranging from 0% to 32% (Figure 2). The pooled prevalence of pCR after neoadjuvant chemoradiation in squamous cell carcinoma was 32% (95% CI: 26%–39%) across 21 studies, ranging from 8% to 66% (Figure 3). For adenocarcinoma, the pooled prevalence of pCR was 6% (95% CI: 1%–12%) after neoadjuvant chemotherapy, across five studies (Figure 4), and 22% (18%–26%) after neoadjuvant chemoradiation across 15 studies (Figure 5). A secondary analysis of all studies, regardless of histologic subtype found a pooled pCR prevalence of 8% (95% CI: 6%–11%) for neoadjuvant chemotherapy (Figure S2) and 29% (95% CI: 26%–32%) for neoadjuvant chemoradiation (Figure S3 **)**.

### 3.2 Associations between study‐ and patient‐level factors and pCR

Exploratory analyses **(**Table 1 **)** found the odds of a pCR were twice as high in single‐arm trials than in randomized trials (OR = 1.99; 95% CI: 1.47–2.70). Regimens that contained three chemotherapeutic agents were associated with higher pCR amongst nCT trial arms (OR = 2.34, 95% CI: 1.40–3.91), but the association was attenuated and compatible with the null hypothesis for nCRT trials arms (OR = 1.26, 95% CI: 0.85–1.86). For adenocarcinoma, neoadjuvant chemo(radiation) regimens that consisted of a platinum‐based chemotherapy with a taxane were associated with a higher rate of pCR than regimens that consisted of platinum‐based chemotherapy with fluorouracil (OR = 3.13; 95% CI: 1.46–6.72). No association was found between the percentage of the study population that was male and pCR (for a 10% increase in male proportion: OR = 0.97, 95% CI: 0.79–1.18). Also, pCR estimates in Asian and non‐Asian countries did not significantly differ (OR = 0.94; 95% CI: 0.66–1.32). Our results also indicated no association with pCR for average age or year of publication (Figures S4 and S5).

**TABLE 1:** Results from unadjusted meta‐regression displaying relationships between study characteristics and pathologic complete response rate.

	Trial type^a^	Regimen count^b^	Regimen type^c^	Induction regimen^d^	Male sex^e^	Trial location^f^
	OR (95% CI)					
Overall	1.99 (1.47–2.70)	0.84 (0.56–1.26)	1.55 (0.96–2.50)	0.73 (0.51–1.06)	0.97 (0.79–1.18)	0.94 (0.66–1.32)
Treatment modality						
NCT	2.70 (1.71–4.28)	2.34 (1.40–3.91)	2.27 (0.79–6.55)		0.78 (0.55–1.08)	1.17 (0.68–2.01)
NCRT	1.27 (0.97–1.66)	1.26 (0.85–1.86)	1.10 (0.78–1.55)	0.73 (0.51–1.06)	0.97 (0.83–1.13)	1.24 (0.93–1.66)
Histology						
Squamous cell carcinoma	1.70 (0.93–3.10)	0.86 (0.44–1.67)	0.98 (0.35–2.72)	0.34 (0.05–2.20)	1.21 (0.79–1.86)	0.65 (0.34–1.24)
Adenocarcinoma	2.00 (1.18–3.41)	0.57 (0.30–1.07)	3.13 (1.46–6.72)	0.99 (0.69–1.43)	0.77 (0.53–1.12)	1.11 (0.46–2.66)

Abbreviations: CI, confidence interval; NCRT, neoadjuvant chemoradiation therapy; NCT, neoadjuvant chemotherapy; OR, odds ratio.

^a^ Single‐arm trials versus randomized trials (reference group).

^b^ Triplet regimen versus doublet regimen (reference group).

^c^ Platinum + taxanes versus platinum + fluorouracil regimen (reference group).

^d^ Induction versus no induction chemoradiation (reference group).

^e^ OR reported for a 10% increase in the proportion of the study population that was male.

^f^ Asian versus non‐Asian regions (reference group).

## 4 DISCUSSION

In this study, we demonstrated that fewer than one‐third of patients diagnosed with nonmetastatic esophageal cancer receiving neoadjuvant chemotherapy or chemoradiation experience pCR. Specifically, we found that trials reporting on patients with squamous cell carcinoma receiving neoadjuvant chemoradiation had the highest pCR rates (32%), while trials reporting on adenocarcinoma receiving neoadjuvant chemotherapy had the lowest pCR rates (6%). Our findings provide synthesized evidence to support that patients with squamous cell carcinoma may be more likely to derive benefit from chemo (radiation) than patients with adenocarcinoma. Collectively, these results support the overall concept that pCR occurs in a minority of patients with esophageal cancer.

Interestingly, we found pCR rates have not improved much over the past three decades. However, in some modality and histology pairs, such as nCRT for squamous cell carcinomas, results from trials testing state‐of‐the‐art neoadjuvant protocols outperformed the time‐pooled summary estimates. Specifically, the CROSS trial used carboplatin and paclitaxel and reported a pCR of 49% in this population, higher than the pooled average of 32%.^7^

The role of pCR in guiding patient–clinician discussions about treatment decision‐making is nuanced and evolving. Prior studies have found that experiencing pCR is associated with longer overall survival than partial response or no response to neoadjuvant treatment.^12^, ^13^, ^15^, ^17^, ^18^ Our work helps to highlight some of the limitations of using pCR alone as a readout for tumor response and surrogate of distant biologic activity. While patients who experience a pCR often have favorable survival outcomes, there is increasing evidence that response in the regional lymph nodes at surgical resection may be a more accurate reflection of biologic activity and presumed micrometastatic control.^109^ However, this requires surgical resection to assess and does not address the increasing desire to devise strategies that may allow avoidance of surgery in some patients. In fact, this approach is being tested in the currently recruiting Neoadjuvant Chemoradiotherapy and Surgery versus Definitive Chemoradiotherapy with Salvage Surgery as Needed (NEEDS) randomized trial, with final results expected in the coming years.^110^ The Surgery As Needed for Oesophageal (SANO) cancer trial is also exploring this approach, with early results suggesting that, amongst those displaying clinical complete response to chemoradiation, active surveillance is noninferior to surgery.^111^, ^112^

Of equal importance to identifying responder patients is the need to understand the larger nonresponder group. By analyzing a large number of trials and reporting the pooled pCR rates, we enhance confidence in the observation that a vast majority of patients do not achieve complete response in the primary tumor. There was a high degree of variability in pCR rates both across treatment modality groups and within groups. In nCT trial arms, patients receiving two chemotherapy agents instead of three were less likely to experience pCR. Notably, the pCR rates were not associated with average patient age or proportion of study population that was male, and did not improve over time in the included trials. While our exploratory analyses may explain some variability in pCR rates, other clinicopathologic features may be driving variability.

An important next step will be enhancing the ability to predict pCR at the individual‐level, both from clinical response and from entirely pretreatment variables. In practice, accurately predicting pCR based on clinical parameters has proven to be a difficult task, with existing work yielding low predictive accuracy.^113^ More accurate prediction of pCR could be instrumental in (1) identifying patients with a high probability of pCR for whom salvage (instead of planned) surgery could be a viable option; (2) identifying patients unlikely to respond to chemo(radiation); and (3) defining populations where novel approaches to intensify (or de‐escalate) neoadjuvant components may be of highest yield.

Our study has several strengths. To our knowledge, this was the first and largest meta‐analysis of pCR rates in trial‐enrolled esophageal carcinoma patients receiving neoadjuvant chemotherapy or chemoradiation. Our literature search strategy was highly sensitive; the broad initial search terms were unlikely to have missed any eligible studies, with over 4500 titles and abstracts screened. The estimates of pCR rate provide context for the expected percentage of patients that are benefitting from neoadjuvant CT or CRT before their surgery. The probabilities gleaned from this meta‐analysis can be used as inputs in decision‐analysis models and cost‐effectiveness analysis. Lastly, with the advent of neoadjuvant immunotherapies, the pooled estimates of PCR from neoadjuvant chemotherapy and chemoradiation provide important benchmarks that serve as inputs into trial design development.

This study is not without limitations. First, we did not include observational studies in our review. Inclusion of observational studies would have further enhanced the sample size and precision of our estimates but concerns about selection bias prohibited their inclusion. In many observational studies, patients with a negative clinical response would be more likely to receive resection and thus also have a documented negative pathologic response. Since our study's objectives were to determine the prevalence of pooled pCR rates and explore relationships between study and patient‐level factors with pCR, we did not examine the relationship between pCR and overall survival. A number of methodologic issues would also have complicated analysis of survival. Only six of 84 studies reported survival stratified by pCR status; studies had a wide difference in length of follow‐up, reported different measures of effect, and calculated survival by randomized assignment as opposed to subset that received surgery (had pCR evaluated). Additionally, we did not extract data on regional lymph node response, an important prognostic variable that may be important to collect in addition to pCR.^109^ We did not collect and analyze data on radiation dose; observational studies are conflicting on whether lower radiation doses are associated with lower pCR rates.^114^, ^115^, ^116^, ^117^

## 5 CONCLUSION

In conclusion, our study provides innovative findings regarding the rates and correlates of pCR among patients with nonmetastatic esophageal cancer receiving neoadjuvant chemotherapy or chemoradiation. In addition, by demonstrating variability in pCR rates according to histology, type of neoadjuvant treatment, and study‐ and patient‐level factors, we highlight the importance of considering the probability that an individual patient will experience pCR when selecting treatment. Future research should focus on developing and validating models to predict the probability of experiencing pCR based on patient‐level variables that could be collected prior to treatment selection.

## AUTHOR CONTRIBUTIONS

**Charles E. Gaber:** Conceptualization (lead); data curation (equal); formal analysis (supporting); investigation (lead); methodology (lead); project administration (lead); supervision (lead); writing – original draft (lead); writing – review and editing (lead). **Jyotirmoy Sarker:** Conceptualization (supporting); data curation (lead); formal analysis (supporting); investigation (supporting); methodology (supporting); project administration (supporting); validation (supporting); visualization (supporting); writing – review and editing (supporting). **Abdullah I. Abdelaziz:** Conceptualization (supporting); data curation (lead); formal analysis (equal); investigation (supporting); methodology (supporting); project administration (supporting); software (lead). **Ebere Okpara:** Data curation (lead); formal analysis (supporting); investigation (supporting); methodology (supporting); visualization (supporting). **Todd A. Lee:** Conceptualization (supporting); investigation (supporting); methodology (supporting); writing – review and editing (equal). **Samuel J. Klempner:** Conceptualization (equal); investigation (equal); writing – original draft (equal); writing – review and editing (equal). **Ryan D. Nipp:** Conceptualization (equal); formal analysis (equal); investigation (equal); methodology (equal); writing – original draft (equal); writing – review and editing (equal).

## FUNDING INFORMATION

No external funding was received for this research.

## CONFLICT OF INTEREST STATEMENT

Dr. Gaber: academic salary support from an educational fellowship from pharmaceutical company AbbVie Inc. Dr. Klempner has no directly relevant conflicts of interest but does perform consulting or advisory board participation for: Astellas, Merck, Bristol‐Myers Squibb, Daiichi‐Sankyo, AstraZeneca, Eli Lilly, Sanofi‐Aventis, Exact Sciences, Servier, Novartis, Coherus Biosciences, Natera, Amgen, and Mersana, reports stock/equity in: Turning Point Therapeutics (ended 6/2022) and Nuvalent (ended 11/2022), has received honoraria from Merck Serono, and research funding from Leap Therapeutics, BeiGene, and Silverback Therapeutics.

### ETHICS STATEMENT

This study was granted exemption from the University of Illinois Chicago Institutional Review Board.

## Supporting information


Data S1.

## Data Availability

Datasets generated and analyzed during the current study are available via supplemental materials online.
