# Supplementary material for: Pathologic complete response in patients with esophageal cancer receiving neoadjuvant chemotherapy or chemoradiation: A systematic review and meta‐analysis
Source: Cancer Med. 2024 Mar 8;13(4):e7076. doi: 10.1002/cam4.7076 (PMC10923050; doi:10.1002/cam4.7076)
Supplement: Supplementary file 1 — Data S1. [file CAM4-13-e7076-s001.docx]

**Supplemental Figure 1.** Breakdown of chemotherapeutic regimens used amongst the 102 trial arms that delivered either neoadjuvant chemotherapy or neoadjuvant chemoradiation

**
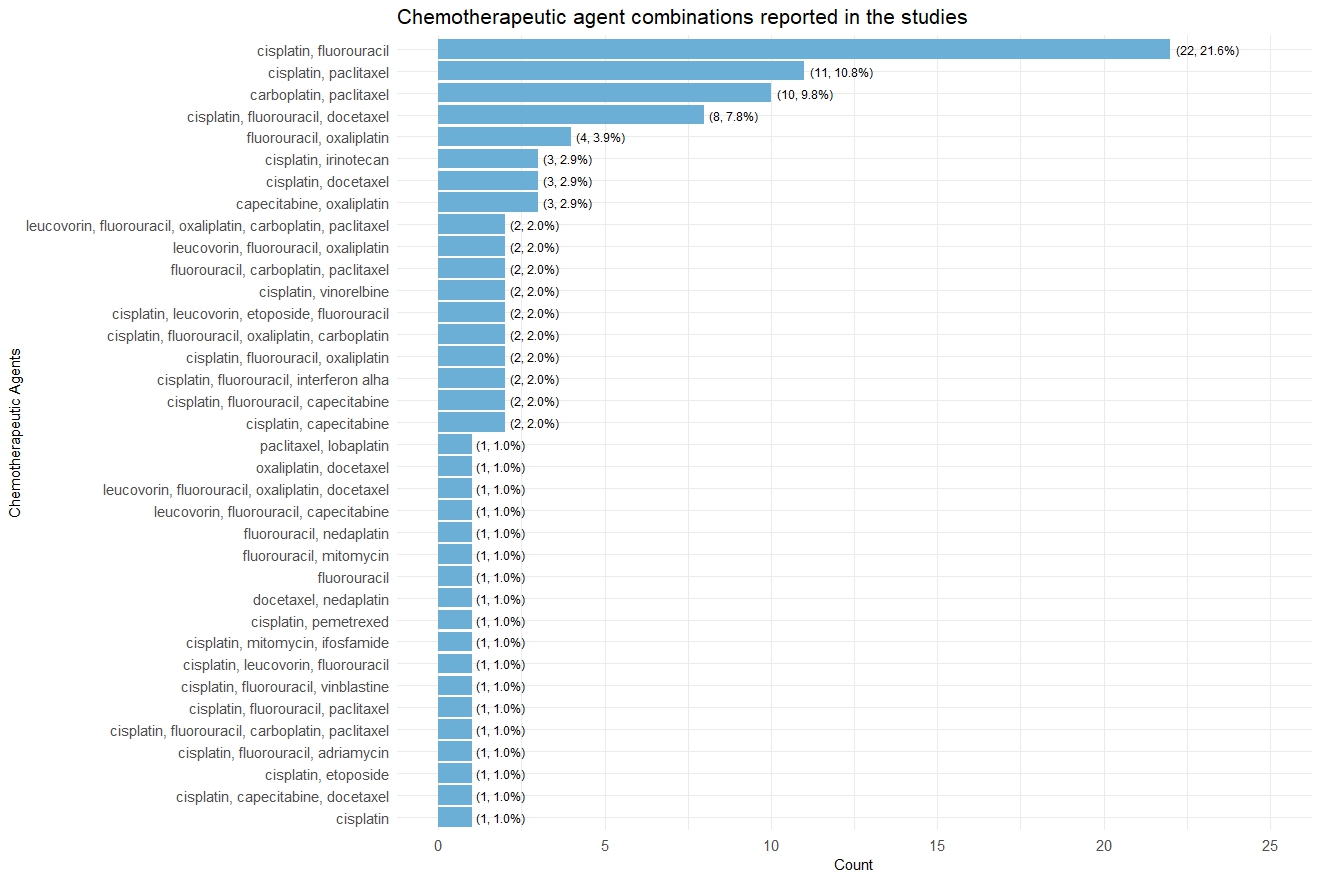
**

**Supplemental Figure 2**. Pathologic complete response amongst trial arms receiving neoadjuvant chemotherapy, all histologic types.

**
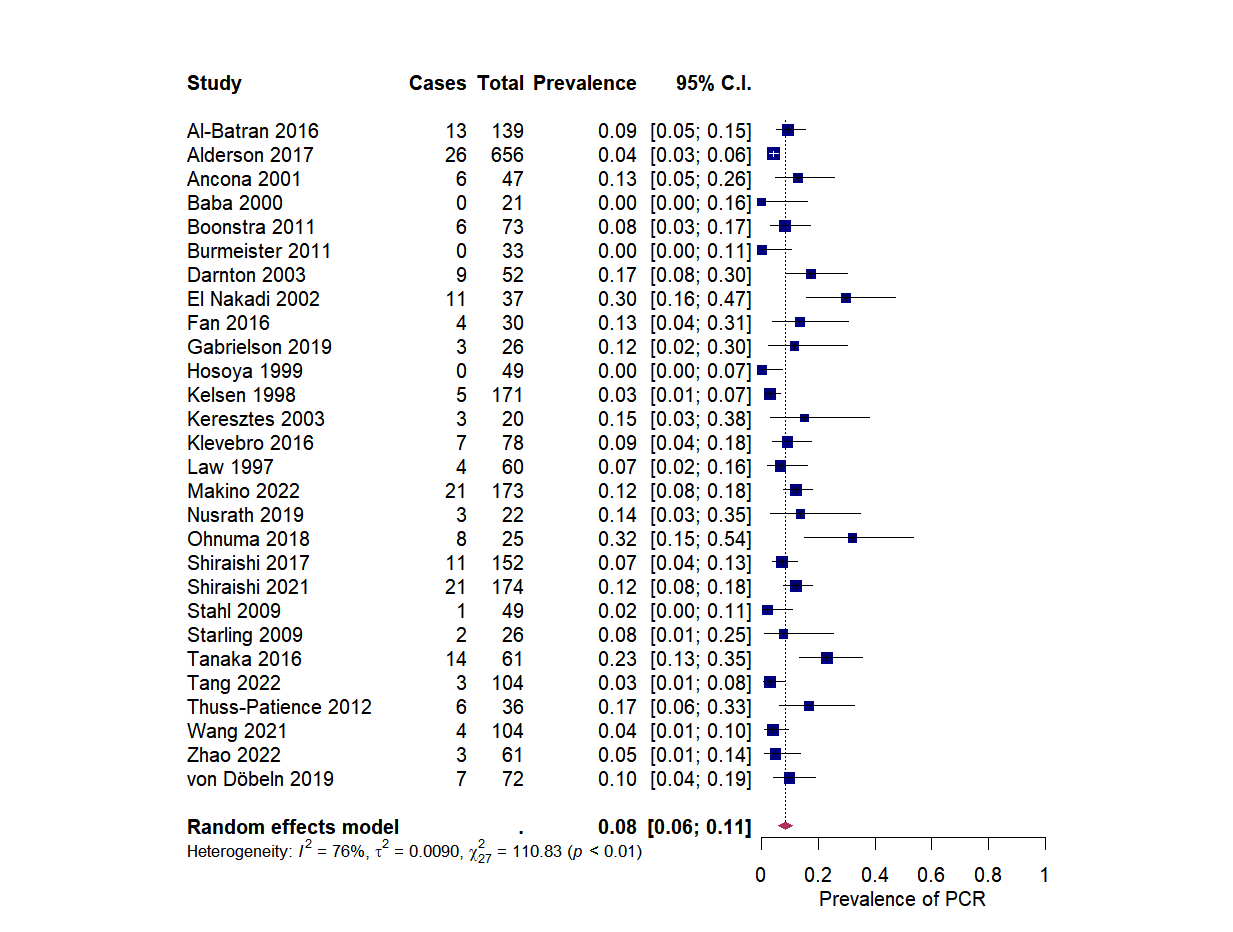
**

**Supplemental Figure 3.** Pathologic complete response amongst trial arms receiving neoadjuvant chemoradiation, all histologic types.
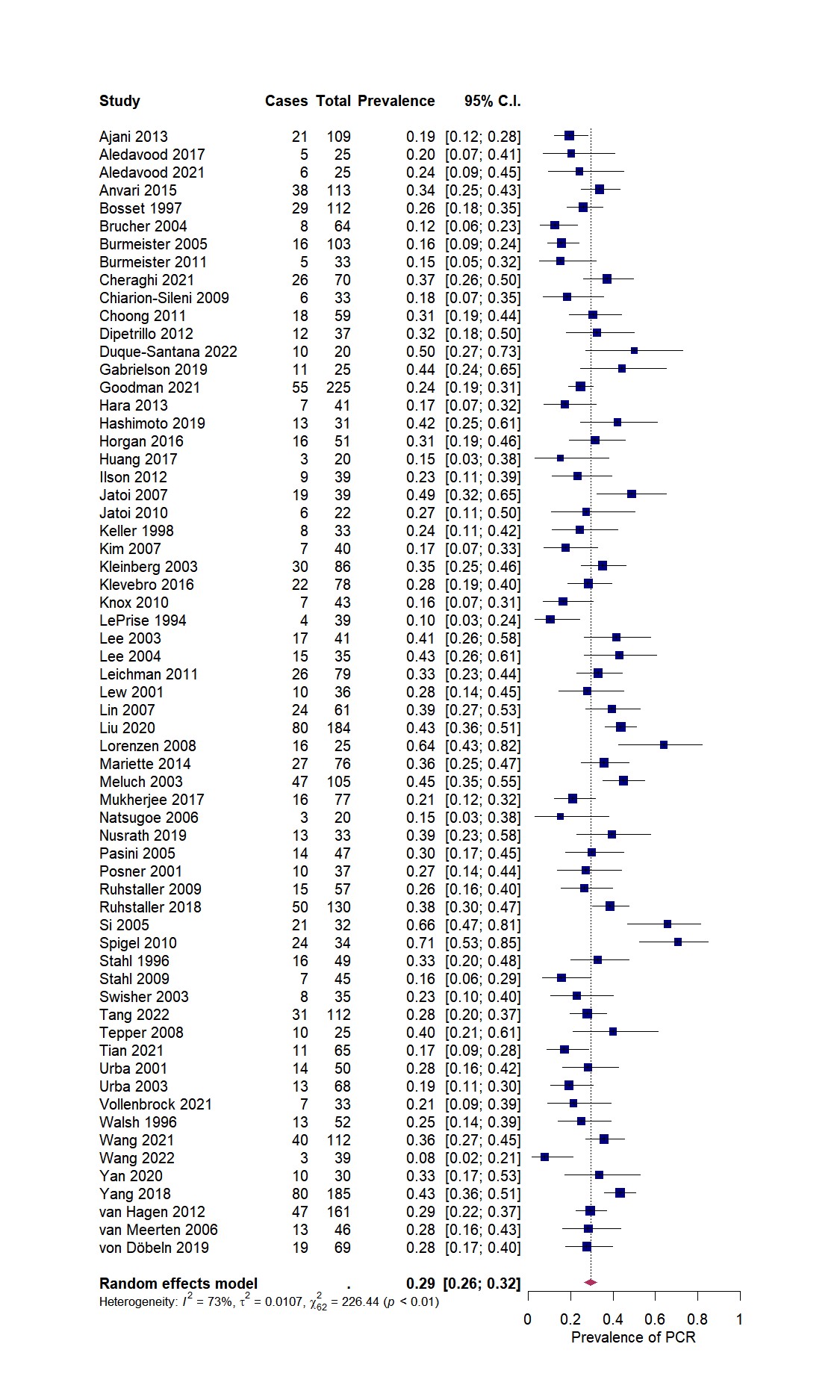


**Supplemental Figure 4.** Meta-regression plots for the prevalence of pathologic complete response by average age of study population, stratified by histology and treatment modality.

**
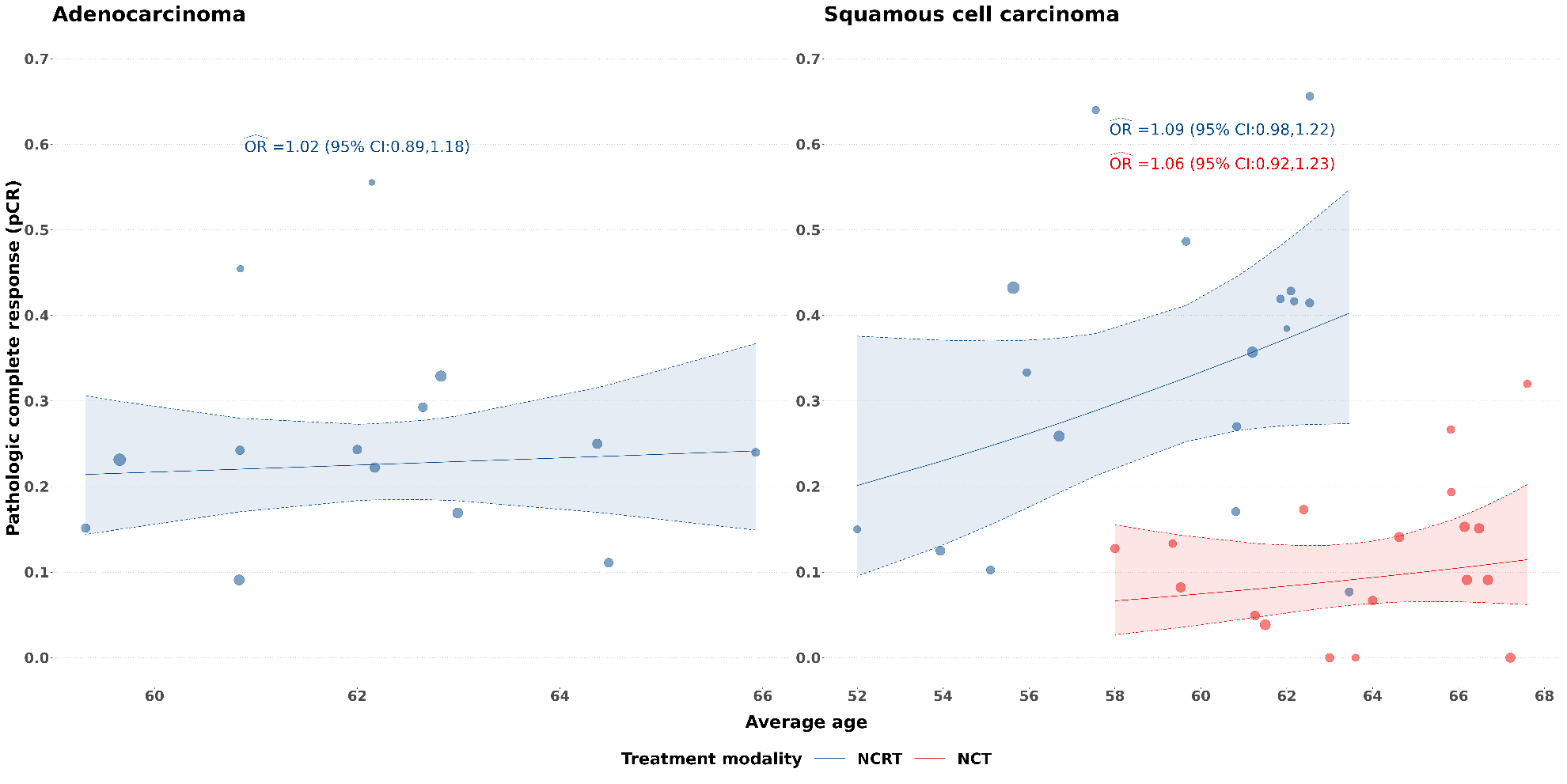
**

**Supplemental Figure 5.** Meta-regression plots for the prevalence of pathologic complete response by year of study publication, stratified by histology and treatment modality.

**
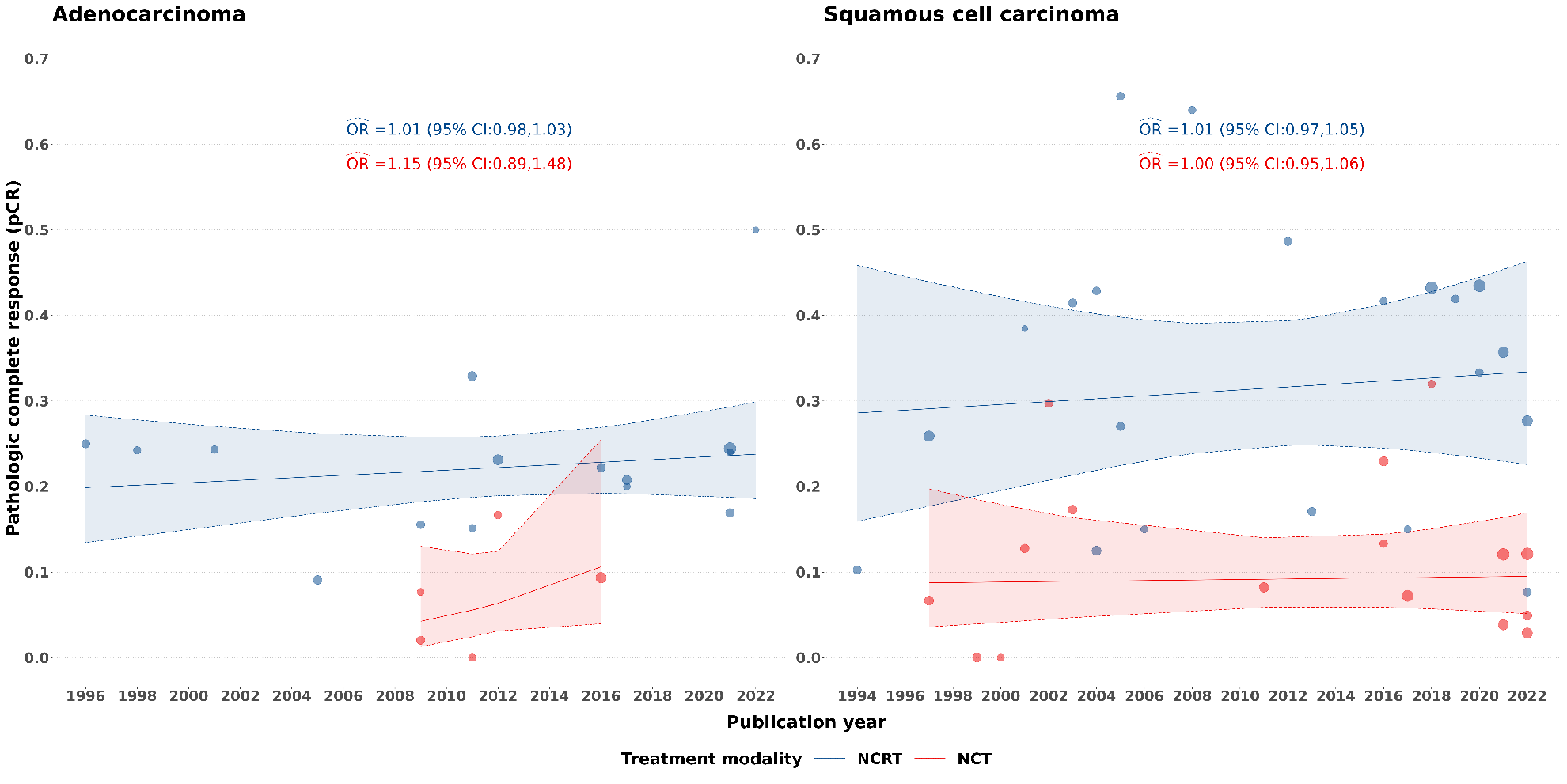
**

**Supplemental Table 1.** PRISMA 2020 checklist for reporting items in manuscript and abstract

# PRISMA 2020 Main Checklist

| **Topic** | **No.** | **Item** | **Location where item is reported** |
| --- | --- | --- | --- |
| **TITLE** |  |  |  |
| **Title** | 1 | Identify the report as a systematic review. | 1 |
| **ABSTRACT** |  |  |  |
| **Abstract** | 2 | See the PRISMA 2020 for Abstracts checklist |  |
| **INTRODUCTION** |  |  |  |
| **Rationale** | 3 | Describe the rationale for the review in the context of existing knowledge. | 3 |
| **Objectives** | 4 | Provide an explicit statement of the objective(s) or question(s) the review addresses. | 4 |
| **METHODS** |  |  |  |
| **Eligibility criteria** | 5 | Specify the inclusion and exclusion criteria for the review and how studies were grouped for the syntheses. | 4-5 |
| **Information sources** | 6 | Specify all databases, registers, websites, organisations, reference lists and other sources searched or consulted to identify studies. Specify the date when each source was last searched or consulted. | 4 |
| **Search strategy** | 7 | Present the full search strategies for all databases, registers and websites, including any filters and limits used. | Supplement |
| **Selection process** | 8 | Specify the methods used to decide whether a study met the inclusion criteria of the review, including how many reviewers screened each record and each report retrieved, whether they worked independently, and if applicable, details of automation tools used in the process. | 5 |
| **Data collection process** | 9 | Specify the methods used to collect data from reports, including how many reviewers collected data from each report, whether they worked independently, any processes for obtaining or confirming data from study investigators, and if applicable, details of automation tools used in the process. | 6 |
| **Data items** | 10a | List and define all outcomes for which data were sought. Specify whether all results that were compatible with each outcome domain in each study were sought (e.g. for all measures, time points, analyses), and if not, the methods used to decide which results to collect. | 6-7 |
|  | 10b | List and define all other variables for which data were sought (e.g. participant and intervention characteristics, funding sources). Describe any assumptions made about any missing or unclear information. | 6 |
| **Study risk of bias assessment** | 11 | Specify the methods used to assess risk of bias in the included studies, including details of the tool(s) used, how many reviewers assessed each study and whether they worked independently, and if applicable, details of automation tools used in the process. | 6 |
| **Effect measures** | 12 | Specify for each outcome the effect measure(s) (e.g. risk ratio, mean difference) used in the synthesis or presentation of results. | 6 |
| **Synthesis methods** | 13a | Describe the processes used to decide which studies were eligible for each synthesis (e.g. tabulating the study intervention characteristics and comparing against the planned groups for each synthesis (item 5)). | 4-5 |
|  | 13b | Describe any methods required to prepare the data for presentation or synthesis, such as handling of missing summary statistics, or data conversions. | NA |
|  | 13c | Describe any methods used to tabulate or visually display results of individual studies and syntheses. | 7 |
|  | 13d | Describe any methods used to synthesize results and provide a rationale for the choice(s). If meta-analysis was performed, describe the model(s), method(s) to identify the presence and extent of statistical heterogeneity, and software package(s) used. | 6-7 |
|  | 13e | Describe any methods used to explore possible causes of heterogeneity among study results (e.g. subgroup analysis, meta-regression). | 7 |
|  | 13f | Describe any sensitivity analyses conducted to assess robustness of the synthesized results. | NA |
| **Reporting bias assessment** | 14 | Describe any methods used to assess risk of bias due to missing results in a synthesis (arising from reporting biases). | NA |
| **Certainty assessment** | 15 | Describe any methods used to assess certainty (or confidence) in the body of evidence for an outcome. | NA |
| **RESULTS** |  |  |  |
| **Study selection** | 16a | Describe the results of the search and selection process, from the number of records identified in the search to the number of studies included in the review, ideally using a flow diagram. | 7, 13 |
|  | 16b | Cite studies that might appear to meet the inclusion criteria, but which were excluded, and explain why they were excluded. | 13 |
| **Study characteristics** | 17 | Cite each included study and present its characteristics. | References (pages 20-30) |
| **Risk of bias in studies** | 18 | Present assessments of risk of bias for each included study. | Supplement |
| **Results of individual studies** | 19 | For all outcomes, present, for each study: (a) summary statistics for each group (where appropriate) and (b) an effect estimate and its precision (e.g. confidence/credible interval), ideally using structured tables or plots. | 14-17 |
| **Results of syntheses** | 20a | For each synthesis, briefly summarise the characteristics and risk of bias among contributing studies. | 7 |
|  | 20b | Present results of all statistical syntheses conducted. If meta-analysis was done, present for each the summary estimate and its precision (e.g. confidence/credible interval) and measures of statistical heterogeneity. If comparing groups, describe the direction of the effect. | 14-17 |
|  | 20c | Present results of all investigations of possible causes of heterogeneity among study results. | 18 |
|  | 20d | Present results of all sensitivity analyses conducted to assess the robustness of the synthesized results. | NA |
| **Reporting biases** | 21 | Present assessments of risk of bias due to missing results (arising from reporting biases) for each synthesis assessed. | NA |
| **Certainty of evidence** | 22 | Present assessments of certainty (or confidence) in the body of evidence for each outcome assessed. | NA |
| **DISCUSSION** |  |  |  |
| **Discussion** | 23a | Provide a general interpretation of the results in the context of other evidence. | 9 |
|  | 23b | Discuss any limitations of the evidence included in the review. | 11 |
|  | 23c | Discuss any limitations of the review processes used. | 11 |
|  | 23d | Discuss implications of the results for practice, policy, and future research. | 11 |
| **OTHER INFORMATION** |  |  |  |
| **Registration and protocol** | 24a | Provide registration information for the review, including register name and registration number, or state that the review was not registered. | 4 |
|  | 24b | Indicate where the review protocol can be accessed, or state that a protocol was not prepared. | 4 |
|  | 24c | Describe and explain any amendments to information provided at registration or in the protocol. | NA |
| **Support** | 25 | Describe sources of financial or non-financial support for the review, and the role of the funders or sponsors in the review. | 1 |
| **Competing interests** | 26 | Declare any competing interests of review authors. | 1 |
| **Availability of data, code and other materials** | 27 | Report which of the following are publicly available and where they can be found: template data collection forms; data extracted from included studies; data used for all analyses; analytic code; any other materials used in the review. | 6 |

#####

# PRIMSA Abstract Checklist

| **Topic** | **No.** | **Item** | **Reported?** |
| --- | --- | --- | --- |
| **TITLE** |  |  |  |
| **Title** | 1 | Identify the report as a systematic review. | Yes |
| **BACKGROUND** |  |  |  |
| **Objectives** | 2 | Provide an explicit statement of the main objective(s) or question(s) the review addresses. | Yes |
| **METHODS** |  |  |  |
| **Eligibility criteria** | 3 | Specify the inclusion and exclusion criteria for the review. | Yes |
| **Information sources** | 4 | Specify the information sources (e.g. databases, registers) used to identify studies and the date when each was last searched. | Yes |
| **Risk of bias** | 5 | Specify the methods used to assess risk of bias in the included studies. | No |
| **Synthesis of results** | 6 | Specify the methods used to present and synthesize results. | Yes |
| **RESULTS** |  |  |  |
| **Included studies** | 7 | Give the total number of included studies and participants and summarise relevant characteristics of studies. | Yes |
| **Synthesis of results** | 8 | Present results for main outcomes, preferably indicating the number of included studies and participants for each. If meta-analysis was done, report the summary estimate and confidence/credible interval. If comparing groups, indicate the direction of the effect (i.e. which group is favoured). | Yes |
| **DISCUSSION** |  |  |  |
| **Limitations of evidence** | 9 | Provide a brief summary of the limitations of the evidence included in the review (e.g. study risk of bias, inconsistency and imprecision). | No |
| **Interpretation** | 10 | Provide a general interpretation of the results and important implications. | Yes |
| **OTHER** |  |  |  |
| **Funding** | 11 | Specify the primary source of funding for the review. | Yes |
| **Registration** | 12 | Provide the register name and registration number. | Yes |

*From:* Page MJ, McKenzie JE, Bossuyt PM, Boutron I, Hoffmann TC, Mulrow CD, et al. The PRISMA 2020 statement: an updated guideline for reporting systematic reviews. MetaArXiv. 2020, September 14. DOI: 10.31222/osf.io/v7gm2. For more information, visit: <www.prisma-statement.org>

**Supplemental Table 2.** Search strategy implemented in Medline, Embase, and CENTRAL.

| Medline (PubMed) | | |
| --- | --- | --- |
|  | #1 NOT #2 NOT #3 | |
|  | #1 | (randomized controlled trial [pt] OR controlled clinical trial [pt] OR randomized [tiab] OR placebo [tiab] OR randomly [tiab] OR trial [tiab] OR clinical trials as topic [mesh:noexp]) AND (((("Esophageal Neoplasms"[Mesh])) OR ((esophag*[tiab] OR oesophag*[tiab] OR gastroesophag*[tiab] OR gastrooesophag*[tiab] OR junction*[tiab]) AND (neoplas*[tiab] OR cancer[tiab] OR cancers[tiab] OR tumor*[tiab] OR malign*[tiab] OR carcinom*[tiab] OR adenocarcinom*[tiab]))) AND (((radio*[All Fields] OR drug*[All Fields] OR chemo*[All Fields] OR radiat*[All Fields] OR therap*[All Fields] OR treatment[All Fields] OR irradiat*[All Fields] OR therapeutics[MeSH] OR therapy[MeSH]) AND (preopera*[All Fields] OR pre-opera*[All Fields] OR adjuvant[All Fields] OR neoadjuvant[All Fields] OR neo-adjuvant[All Fields] OR peri-opera*[All Fields] OR periopera*[All Fields] OR pre-surg*[All Fields] OR presurg*[All Fields])) OR ((("Neoadjuvant Therapy"[Mesh]) OR ("Antineoplastic Combined Chemotherapy Protocols"[Mesh])) OR ("Chemotherapy, Adjuvant"[Mesh])))) AND ((english[Filter]) AND (1992:2022[pdat])) |
|  | #2 | Review[pt] OR Systematic Review[pt] OR Meta-Analysis[pt] |
|  | #3 | animals [mh] NOT humans [mh] |
|  |  |  |
| Embase | | |
|  |  | ('esophageal gastrointestinal stromal tumor'/exp OR 'esophag*':ti,ab OR 'oesophag*':ti,ab OR 'gastroesophag*':ti,ab OR 'junction*':ti,ab OR 'neoplas*':ti,ab OR 'cancer':ti,ab OR 'tumor*':ti,ab OR 'malign*':ti,ab OR 'carcinom*':ti,ab OR 'adenocarcinom*':ti,ab ) AND ('controlled clinical trial'/exp OR 'randomized':ti,ab OR 'placebo':ti,ab OR 'randomly':ti,ab OR 'trial':ti,ab) AND ('cancer therapy'/exp OR 'radio' OR 'drug' OR 'chemo*' OR 'radiat*' OR 'treatment' OR 'therap*' OR 'therapy') AND ('neoadjuvant chemotherapy'/exp OR 'cancer adjuvant therapy'/exp OR 'antineoplastic protocol'/exp OR 'preopera*' OR 'pre-opera*' OR 'adjuvant' OR 'neoadjuvant' OR 'neo-adjuvant' OR 'peri-opera*' OR 'periopera*' OR 'pre-surg*' OR 'presurg*') |
| CENTRAL | | |
|  |  | #1 MeSH descriptor: [Randomized Controlled Trial] explode all trees  #2 (random*):ti,ab,kw OR (placebo):ti,ab,kw OR (trial):ti,ab,kw  #3 MeSH descriptor: [Esophageal Neoplasms] explode all trees  #4 (esophag*):ti,ab,kw OR (oesophag*):ti,ab,kw OR (gastroesophag*):ti,ab,kw OR (gastrooesophag*):ti,ab,kw OR (junction*):ti,ab,kw  #5 (neoplas*):ti,ab,kw OR (cancer*):ti,ab,kw OR (tumor*):ti,ab,kw OR (malign*):ti,ab,kw OR (carcinom*):ti,ab,kw  #6 (adenocarcinom*):ti,ab,kw  #7 (radio*) OR (drug*) OR (chemo*) OR (radiat*) OR (therap*)  #8 (irradiat*)  #9 MeSH descriptor: [Therapeutics] explode all trees  #10 (preopera*) OR (pre-opera*) OR (adjuvant) OR (neoadjuvant) OR (neo-adjuvant)  #11 (peri-opera*) OR (periopera*) OR (pre-surg*) OR (presurg*)  #12 MeSH descriptor: [Neoadjuvant Therapy] explode all trees  #13 MeSH descriptor: [Antineoplastic Combined Chemotherapy Protocols] explode all trees  #14 MeSH descriptor: [Chemotherapy, Adjuvant] explode all trees  #15 MeSH descriptor: [Systematic Reviews as Topic] explode all trees  #16 MeSH descriptor: [Meta-Analysis as Topic] explode all trees  #17 MeSH descriptor: [Randomized Controlled Trials as Topic] explode all trees  #18 #1 OR #2 OR #17  #19 #5 OR #6  #20 #4 and #19  #21 #3 OR #20  #22 #7 OR #8 OR #9  #23 #10 OR #11 OR #12 OR #13 OR #14  #24 #15 OR #16  #25 (#1 OR #2 OR #17) AND (#3 OR (#4 AND (#5 OR #6))) AND (#7 OR #8 OR #9) AND (#10 OR #11 OR #12 #13 OR #14) |

**Supplemental Table 3**. Quality assessment questions modified from JBI Critical Appraisal Checklist for Studies Reporting Prevalence Data

|  | **Quality assessment questions** | **Criteria for different response** |
| --- | --- | --- |
| 1 | Was the sample representative of the target population? | This question is about the generalizability of the prevalence estimate to our broad target population of esophageal cancer patients with non-metastatic tumors. If the study eligibility criteria was restricted to a specific age range (e.g. only those 70 and younger) or sex (e.g. only males), or narrow stage-grouping within non-metastatic disease (e.g. only stages IIA-IIB) then the answer is "No". |
| 2 | Were study participants recruited in an appropriate way? | For every study, this one will be ‘not applicable’. |
| 3 | Was the sample size adequate? | For every study, this one will be ‘not applicable’. |
| 4 | Were the study subjects and setting described in detail? | Certain diseases or conditions vary in prevalence across different geographic regions and populations (e.g. women vs. men, socio-demographic variables between countries). Has the study sample been described in sufficient detail so that other researchers can determine if it is comparable to the population of interest to them? |
| 5 | Was the data analysis conducted with sufficient coverage of the identified sample? | If a substantial (>20%) proportion of individuals who were randomized to surgery did not receive surgery (e.g. too sick), this would be "No". |
| 6 | Were objective, standard criteria used for the measurement of the condition? | Yes = Used Mandard, Becker, Chirieac, or Stewart systems for defining PCR; No = just called it PCR or said no residual tumor |
| 7 | Was the condition measured reliably? | Yes if the paper mentions the surgical specimen was assessed by a pathologist, "No" if it was clearly determined by somebody else (oncologist), "Unclear" if it doesn't mention who performed it. |
| 8 | Was there appropriate statistical analysis? | For every study, this one will be ‘not applicable’. |
| 9 | Are all important confounding factors/ subgroups/differences identified and accounted for? | If the study is restricted to one histologic subtype (e.g. adenocarcinomas), then this is not applicable. If it allows more than one subtype then answer "Yes" if it reports PCR stratified by subtype, "No" if it only gives PCR for the all patients in an arm overall. Since it has to be one of these, there is no "unclear" response. |
| 10 | Were subpopulations identified using objective criteria? | For every study, this one will be ‘not applicable’. |

**Supplemental Table 4**. Study-level results of quality assessment questions

| **Study ID** | **1** | **2** | **3** | **4** | **5** | **6** | **7** | **8** | **9** | **10** |
| --- | --- | --- | --- | --- | --- | --- | --- | --- | --- | --- |
| Ajani 2013 | Y | NA | NA | Y | Y | Y | U | NA | N | NA |
| Al-Batran 2016 | U | NA | NA | Y | Y | Y | Y | NA | NA | NA |
| Alderson 2017 | Y | NA | NA | Y | Y | Y | Y | NA | N | NA |
| Aledavood 2017 | Y | NA | NA | Y | N | N | Y | NA | NA | NA |
| Aledavood 2021 | N | NA | NA | Y | N | N | Y | NA | NA | NA |
| Ancona 2001 | Y | NA | NA | Y | Y | N | U | NA | NA | NA |
| Anvari 2015 | N | NA | NA | N | Y | Y | U | NA | N | NA |
| Baba 2000 | N | NA | NA | U | Y | N | Y | NA | NA | NA |
| Boonstra 2011 | U | NA | NA | U | Y | N | U | NA | NA | NA |
| Bosset 1997 | N | NA | NA | Y | Y | Y | U | NA | NA | NA |
| Brücher 2004 | U | NA | NA | N | Y | Y | Y | NA | NA | NA |
| Burmeister 2005 | Y | NA | NA | N | Y | N | U | NA | Y | NA |
| Burmeister 2011 | U | NA | NA | Y | Y | N | N | NA | NA | NA |
| Cheraghi 2021 | U | NA | NA | N | Y | N | Y | NA | N | NA |
| Chiarion-Sileni 2009 | Y | NA | NA | Y | Y | N | U | NA | N | NA |
| Choong 2011 | Y | NA | NA | U | U | N | U | NA | N | NA |
| Darnton 2003 | U | NA | NA | N | Y | N | Y | NA | NA | NA |
| Dipetrillo 2012 | U | NA | NA | N | Y | N | N | NA | N | NA |
| Duque-Santana 2022 | U | NA | NA | Y | Y | N | U | NA | NA | NA |
| ElNakadi 2002 | U | NA | NA | N | Y | N | U | NA | NA | NA |
| Fan 2016 | Y | NA | NA | Y | Y | Y | Y | NA | NA | NA |
| Gabrielson 2019 | U | NA | NA | U | U | N | Y | NA | N | NA |
| Goodman 2021 | Y | NA | NA | U | Y | N | Y | NA | NA | NA |
| Hara 2013 | U | NA | NA | Y | Y | N | U | NA | NA | NA |
| Hashimoto 2019 | U | NA | NA | Y | Y | N | U | NA | NA | NA |
| Horgan 2016 | U | NA | NA | U | Y | Y | Y | NA | N | NA |
| Hosoya 1999 | N | NA | NA | N | Y | Y | U | NA | NA | NA |
| Huang 2017 | N | NA | NA | Y | Y | Y | Y | NA | Y | NA |
| Ilson 2012 | Y | NA | NA | Y | Y | N | U | NA | N | NA |
| Jatoi 2007 | U | NA | NA | N | Y | N | U | NA | N | NA |
| Jatoi 2010 | Y | NA | NA | Y | Y | N | U | NA | N | NA |
| Keller 1998 | U | NA | NA | U | U | N | U | NA | NA | NA |
| Kelsen 1998 | Y | NA | NA | N | Y | N | U | NA | N | NA |
| Keresztes 2003 | Y | NA | NA | Y | Y | N | U | NA | Y | NA |
| Kim 2007 | Y | NA | NA | Y | Y | N | U | NA | N | NA |
| Kleinberg 2003 | Y | NA | NA | Y | Y | N | U | NA | Y | NA |
| Klevebro 2016 | Y | NA | NA | Y | Y | N | Y | NA | N | NA |
| KNx 2010 | Y | NA | NA | U | Y | Y | Y | NA | N | NA |
| Law 1997 | Y | NA | NA | U | Y | N | U | NA | Y | NA |
| Lee 2003 | Y | NA | NA | Y | Y | N | U | NA | Y | NA |
| Lee 2004 | Y | NA | NA | Y | Y | N | U | NA | Y | NA |
| Leichman 2011 | Y | NA | NA | U | Y | N | Y | NA | Y | NA |
| LePrise 1994 | Y | NA | NA | U | Y | N | U | NA | Y | NA |
| Lew 2001 | Y | NA | NA | U | Y | N | U | NA | N | NA |
| Lin 2007 | N | NA | NA | Y | N | N | U | NA | N | NA |
| Liu 2020 | Y | NA | NA | Y | Y | N | U | NA | NA | NA |
| Lorenzen 2008 | Y | NA | NA | Y | Y | Y | U | NA | NA | NA |
| MakiN 2022 | Y | NA | NA | Y | Y | N | U | NA | NA | NA |
| Mariette 2014 | Y | NA | NA | Y | Y | Y | U | NA | N | NA |
| Meluch 2003 | Y | NA | NA | Y | Y | N | U | NA | Y | NA |
| Mukherjee 2017 | Y | NA | NA | U | Y | Y | Y | NA | NA | NA |
| Natsugoe 2006 | N | NA | NA | N | Y | N | U | NA | NA | NA |
| Nusrath 2019 | U | NA | NA | Y | U | U | U | NA | NA | NA |
| Ohnuma 2018 | Y | NA | NA | Y | Y | Y | U | NA | NA | NA |
| Pasini 2005 | U | NA | NA | U | Y | N | U | NA | NA | NA |
| Posner 2001 | Y | NA | NA | U | Y | N | U | NA | N | NA |
| Ruhstaller 2009 | Y | NA | NA | Y | Y | Y | Y | NA | Y | NA |
| Ruhstaller 2018 | Y | NA | NA | Y | Y | Y | U | NA | N | NA |
| Shiraishi 2017 | Y | NA | NA | Y | Y | N | U | NA | NA | NA |
| Shiraishi 2021 | Y | NA | NA | Y | Y | N | U | NA | NA | NA |
| Si 2005 | N | NA | NA | Y | Y | N | Y | NA | NA | NA |
| Spigel 2010 | Y | NA | NA | Y | Y | N | U | NA | N | NA |
| Stahl 1996 | Y | NA | NA | U | Y | N | U | NA | N | NA |
| Stahl 2009 | N | NA | NA | Y | Y | Y | U | NA | NA | NA |
| Starling 2009 | Y | NA | NA | Y | N | N | Y | NA | NA | NA |
| Swisher 2003 | N | NA | NA | N | Y | N | Y | NA | N | NA |
| Tanaka 2016 | N | NA | NA | Y | Y | N | U | NA | NA | NA |
| Tanaka 2016 | Y | NA | NA | Y | N | N | U | NA | NA | NA |
| Tang 2022 | Y | NA | NA | N | N | N | U | NA | NA | NA |
| Tepper 2008 | N | NA | NA | Y | Y | N | Y | NA | N | NA |
| Tian 2021 | Y | NA | NA | Y | Y | N | N | NA | NA | NA |
| Thuss-Patience 2012 | Y | NA | NA | Y | Y | Y | Y | NA | NA | NA |
| Urba 2001 | N | NA | NA | U | N | N | U | NA | Y | NA |
| Urba 2003 | Y | NA | NA | U | Y | N | U | NA | N | NA |
| vanHagen 2012 | Y | NA | NA | Y | Y | Y | U | NA | Y | NA |
| vanMeerten 2006 | Y | NA | NA | Y | Y | N | U | NA | N | NA |
| Vollenbrock 2021 | N | NA | NA | N | Y | Y | Y | NA | N | NA |
| von Döbeln 2019 | N | NA | NA | Y | N | N | U | NA | N | NA |
| Walsh 1996 | Y | NA | NA | U | Y | N | U | NA | NA | NA |
| Wang 2021 | N | NA | NA | Y | Y | N | U | NA | N | NA |
| Wang 2022 | N | NA | NA | Y | Y |  | U | NA | NA | NA |
| Yan 2020 | N | NA | NA | U | Y | N | N | NA | NA | NA |
| Yang 2018 | N | NA | NA | Y | Y | Y | U | NA | NA | NA |
| Zhao 2022 | N | NA | NA | U | Y | N | U | NA | NA | NA |
